# Cardiac phenotype in hereditary transthyretin amyloidosis: correlations between fibril types and 99mTc-DPD uptake

**DOI:** 10.1038/s41598-026-43816-x

**Published:** 2026-03-16

**Authors:** Viktor Löfbacka, Jonas Wixner, Per Westermark, Justina Damjanovic Vesterlund, Intissar Anan, Björn Pilebro

**Affiliations:** 1 Heart Centre, Department of Clinical Medicine, Umeå University, Umeå, Sweden; 2 Department of Clinical Medicine, Umeå University, Umeå, Sweden; 3 Department of Immunology, Genetics and Pathology, Uppsala University, Uppsala, Sweden

**Keywords:** Transthyretin amyloidosis, fibril type, Cardiomyopathy, 99mTc-DPD, Val30Met, Cardiology, Diseases, Medical research

## Abstract

Variant transthyretin amyloidosis is a systemic disease. In Sweden, the Val30Met variant is the most prevalent. Val30Met presents in two phenotypes: an early-onset form dominated by polyneuropathy and a late-onset form frequently accompanied by cardiomyopathy. These phenotypes are associated with two amyloid fibril types. Type A fibrils, contain both fragmented and full-length transthyretin, whereas type B fibrils contain only full-length transthyretin. Fibril type has been linked to differences in cardiac tracer uptake on 99mTc-DPD scintigraphy. A total of 152 patients with confirmed variant transthyretin amyloidosis evaluated at Umeå University Hospital, Sweden, were included. Age at disease onset and cardiac involvement, investigated by echocardiography, Troponin-T, and NT-proBNP, were assessed in relation to fibril type in abdominal fat and scintigraphic findings. Eighty-five patients had type A fibrils and sixty-seven had type B fibrils. Type A patients were older at diagnosis and had pathologic scintigraphies and more severe cardiac involvement. A subset of type B patients (15%) exhibited cardiac tracer uptake and had cardiac characteristics and age at disease onset similar to those with type A fibrils. Even though there was a strong correlation with findings in abdominal fat pad biopsies, results from 99mTc-DPD scintigraphy correlated better with clinical phenotype.

## Introduction

Transthyretin amyloidosis (ATTR) is a systemic disease affecting multiple organ systems. The most common symptomatic organ manifestations of ATTR amyloidosis are polyneuropathy and cardiomyopathy^1–3^. ATTR amyloidosis occurs in an autosomal dominant hereditary form (ATTRv) and can also be acquired as wild-type transthyretin (ATTRwt) amyloidosis. There are over 140 known variants in the *TTR* gene^4^. The most common variant globally, as well as in Sweden, is the Val30Met (*p. Val50Met*) variant, ^2^.

In the ATTR Val30Met population, two rather distinct phenotypes have been observed: an early-onset and a late-onset phenotype. The early-onset type generally affects younger individuals and has polyneuropathy as the predominant clinical manifestation. The late-onset type manifests at an older age and is associated with both polyneuropathy and amyloid cardiomyopathy^2^. An age of onset before or after 50 years of age has often been used as a cut-off between the two groups^2,4^.

Tissue analyses have shown that differences in amyloid fibril composition are associated with these phenotypic differences. Fibril type A, associated with late-onset disease, consists of a combination of C-terminal TTR fragments starting around position 50 and full-length TTR. In contrast, fibril type B, associated mainly with early-onset disease, consists only of full-length TTR^5,6^. It has therefore been suggested that ATTR fibrils may form through two distinct pathways: one in which amyloid is formed from full-length TTR, and one in which proteolytic cleavage of native TTR is associated with amyloid formation^6^. Type B fibrils have thus far been identified in only two TTR variants, Val30Met and Tyr114Cys (*p. Tyr134Cys*), while all other variants, as well as ATTRwt, are associated with deposition of type A fibrils. However, to our knowledge, only 29 of the known variants causing ATTR amyloidosis have been analysed regarding fibril type^2,6,7^.

Transthyretin amyloid cardiomyopathy (ATTR-CM) affects the heart by causing a stiffening of the heart with a restrictive cardiomyopathy and heart failure as a result, as well as conduction disturbances and arrhythmias^8,9^. Toxic effects of ATTR aggregates, as well as reduced myocardial perfusion, have been suggested in the pathogenesis^10,11^.

Robust and easily accessible echocardiographic and biochemical findings indicating cardiomyopathy and heart failure in ATTR-CM include increased interventricular septum thickness at end-diastole (IVSd), as well as elevated Troponin-T - and N-terminal pro-B-type natriuretic peptide (NT-proBNP) levels. Patients with ATTR-CM also often present with heart failure characterised by preserved ejection fraction^12,13^.

When suspected, bone scintigraphy (in Sweden using 99mTc-3,3-diphosphono-1,2-propanodicarboxylic acid (99mTc-DPD)) can be utilised as part of a non-invasive diagnostic algorithm to identify ATTR-CM ^8^. For purposes of ATTR-CM diagnostics, a positive scintigraphy is defined as an uptake grade of ≥ 2 on a 4-point scale from 0 to 3 according to the Perugini grading, while grade 1 uptake is considered inconclusive^14^.

Although bone scintigraphy is highly sensitive for diagnosing ATTR-CM ^9^, cases of ATTRv amyloidosis with negative bone scintigraphy scans have been reported, particularly in carriers of Phe64Leu and Val30Met variants^15,16^. We previously demonstrated, using PET with Pittsburgh compound B, that cardiac TTR amyloid deposits can be present irrespective of fibril type and 99mTc-DPD results^17^.

In the present study, we sought to investigate, in a larger cohort, the relationship between ATTR fibril composition and 99mTc-DPD scintigraphy uptake, as well as indicators of cardiac involvement assessed by echocardiography and cardiac biomarkers.

## Materials and methods

### Patient selection

Between 2012 and 2020, 316 patients with ATTRv amyloidosis were evaluated at the Amyloidosis Centre, Umeå University Hospital, Umeå, Sweden. Patients were eligible if they had both 99mTc-DPD scintigraphy and fibril composition analysis of abdominal fat pad biopsies. Patients who had not undergone 99mTc-DPD scintigraphy locally (*n* = 89), had uninterpretable or negative abdominal fat tissue biopsies (*n* = 73), or were heart transplanted prior to 99mTc-DPD scintigraphy (*n* = 2) were excluded from analysis. Thus, 152 patients were included in the analysis.

### Patient characteristics

Patient characteristics were obtained through a review of medical records. The data collected included sex and age at diagnosis. Information on hypertension was also recorded, with hypertension defined either as a documented clinical diagnosis or the presence of ≥ 2 blood pressure measurements showing systolic blood pressure > 140 mmHg or diastolic blood pressure > 90 mmHg at diagnosis.

### Histopathological analysis

All tissue biopsies were obtained from subcutaneous abdominal fat since the amount of amyloid in cardiac biopsies is insufficient for the determination of ATTR fibril type. Analyses were performed at the Department of Pathology, Akademiska Hospital, Uppsala, Sweden, according to a previously described method^18^. To detect full-length as well as C-terminal TTR fragments, a western blot analysis with a polyclonal rabbit antiserum against TTR50-127 was used^5,19^. The fibril type was assessed as type A fibril if any amount of truncated TTR, in addition to a characteristic band appearing between the TTR monomer and dimer bands, was found.

In cases where fibril typing did not follow the earlier established phenotypic pattern, new blinded histopathological analyses were performed for re-evaluation.

### Amyloid load scoring

The relative amount of amyloid in the squeeze preparations, stained with Congo red and examined under a polarisation microscope, was semi-quantitatively scored as follows: 0 = no amyloid, 1 = one or a few deposits in several tissue fragments, 2 = many small deposits in several tissue fragments, and 3 = larger, widely spread deposits. It should be noted that type B deposits typically appeared more indistinct, in comparison with type A, where the amyloid usually formed sharply limited bodies.

### 99mTc-DPD scintigraphy

All patients underwent scintigraphy at Umeå University Hospital using a hybrid single-photon emission computed tomography (SPECT)-CT gamma camera (General Electric Medical Systems, Infinia Hawkeye) equipped with a Low Energy High Resolution (LEHR) collimator after intravenous injection of 740 MBq of 99mTc-DPD. Whole body planar images were acquired 5 min and 3 h post-injection in a 256 × 1024 matrix. Cardiac SPECT-CT imaging was subsequently performed with a 128 × 128 matrix in 30 projections. Reconstruction employed an iterative algorithm (OSEM, 3 iterations, 10 subsets) with CT-based attenuation correction. Image fusion and reconstruction were performed using the GE Celeries workstation, and CT volume data were reconstructed into 5 mm slices.

For this study, cardiac uptake of 99mTc-DPD was visually scored in planar images using the Perugini grading system (grades 0–3), where: Grade 0: Absence of cardiac uptake with normal bone uptake; Grade 1: Mild cardiac uptake less than bone uptake; Grade 2: Moderate cardiac uptake with attenuated bone uptake and Grade 3: Strong cardiac uptake with minimal or absent bone uptake.

This visual scoring method has been previously validated for the diagnosis of cardiac amyloidosis^20,21^.

### Echocardiography

All echocardiographic examinations were done by experienced sonographers at Umeå University Hospital, following the guidelines of the American Society of Echocardiography^22^. Standard measurements were obtained to evaluate cardiac structure and function. Left ventricular ejection fraction (LVEF) was categorised into the following groups for analysis: LVEF ≥ 50%; LVEF 40–49%; LVEF < 40%, and unavailable LVEF (due to incomplete or non-assessable data).

### Cardiac biomarkers and Estimated Glomerular Filtration Rate (eGFR)

Cardiac biomarkers, including Troponin-T and NT-proBNP, were analysed in the clinical chemistry laboratory at Umeå University Hospital using a Cobas 800 analyser (Roche Diagnostics Scandinavia). Troponin-T was measured using the high-sensitivity STAT assay, and NT-proBNP levels were determined using STAT assay, both following the manufacturer´s protocols. Estimated glomerular filtration rate (eGFR) was calculated using the Chronic Kidney Disease Epidemiology Collaboration (CKD-EPI) equation based on serum creatinine measurements, as per standard clinical practice.

### Statistical analyses

Statistical analyses were performed using IBM SPSS Statistics^®^ (version 29). Descriptive data were presented separately for the two fibril composition groups. Differences between groups were assessed with the Chi-square test. Continuous variables were assessed using Mann-Whitney U tests, after normality was excluded by the Shapiro-Wilk Test. Comparisons between patients with type B fibril composition and positive 99mTc-DPD scintigraphy uptake were also performed using the Mann-Whitney U test, due to the small sample size.

The distribution of tissue amyloid load grades between groups was evaluated using Mann–Whitney U test. Correlations between amyloid load grade and Perugini grade, as well as continuous variables reflecting cardiac involvement, were evaluated using Spearman’s rho, due to the ordinal nature of the grades and non-normal distribution of the variables.

Continuous variables are presented as median and interquartile range (IQR). A p-value < 0.05 was considered statistically significant.

### Ethics

The study was conducted according to the Helsinki Declaration, and all patients had given written informed consent to participate in the study. Study procedures were approved by the Regional Ethical Review Board in Umeå (Dnr: 2013-407-31 M).

## Results

Of the 152 patients included in the study, 85 had fibril type A and 67 fibril type B. The most common variant was Val30Met (89%), present in all patients with type B fibrils and in 69 patients (81%) with type A fibrils. Additional variants in the type A group included His88Arg (*n* = 7), Phe33Leu (*n* = 2). Other variants included Ala45Gly, Ala45Ser, Thr60Ala, Val122Ile, Ala97Ser, Glu89Lys, and Glu54Leu (*n* = 1 each).

### Comparisons between type A and type B fibril composition

As shown in Table 1, patients with fibril type A composition were significantly older at the time of 99mTc-DPD scintigraphy and at diagnosis compared to those with type B fibril composition (*p* < 0.001, respectively). Type A patients also exhibited significantly greater IVSd, higher Troponin-T levels, and NT-proBNP levels compared to type B patients (*p* < 0.001 for all). A higher prevalence of negative 99mTc-DPD scans was observed in the type B (85%) compared with none in the type A group (*p* < 0.001).

**Table 1:** Patient characteristics by fibril type.

Category	Fibril type A (*n* = 85)					Fibril type B (*n* = 67)					*p*-value
	*n* (%)	Min	Max	Median	IQR	*n* (%)	Min	Max	Median	IQR	
**Sex (male)**	57 (67.1%)					42 (62.7%)					0.574
**Age at diagnosis**	85 (100.0%)	39	87	69	8	67 (100.0%)	28	77	55	25	< 0.001
**Age at 99mTc-DPD**	85 (100.0%)	40	87	70	8	67 (100.0%)	29	80	59	27	< 0.001
**Perugini grade**											
Perugini grade 0	0 (0.0%)					57 (85.1%)					< 0.001
Perugini grade 1	3 (3.5%)					1 (1.5%)					0.436
Perugini grade 2	22 (25.9%)					6 (9.0%)					0.008
Perugini grade 3	60 (70.6%)					3 (4.5%)					< 0.001
**Amyloid load**											
Amyloid load grade 1	30 (35.3%)					13 (19.4%)					0.031
Amyloid load grade 2	24 (28.2%)					24 (35.5%)					0.318
Amyloid load grade 3	28 (32.9%)					27 (40.3%)					0.349
Amyloid load not analysed	3 (3.5%)					3 (3.5%)					0.766
**Val30Met**	69 (81.2%)					67 (100.0%)					< 0.001
**IVSd**	84 (98.8%)	7	25	16	6	67 (100.0%)	7	26	11	3	< 0.001
IVSd > 12 mm	73 (85.9%)					20 (29.9%)					< 0.001
IVSd ≤ 12 mm	11 (12.9%)					47 (70.1%)					< 0.001
IVSd not analysed	1 (1.2%)					0 (0.0%)					0.373
**LVEF**											
LVEF ≥ 50%	53 (62.4%)					56 (83.6%)					0.004
LVEF 40–49%	9 (10.6%)					1 (1.5%)					0.025
LVEF < 40%	9 (10.6%)					2 (3.0%)					0.072
LVEF not analysed	14 (16.5%)					8 (11.9%)					0.431
**Troponin-T (ng/l)**	80 (94.1%)	4	532	30	31	58 (86.6%)	4	169	11	17	< 0.001
**NT-proBNP (ng/l)**	84 (98.8%)	25	10,085	861.5	2581	63 (94.0%)	10	9210	162	446	< 0.001
**eGFR**	85 (100.0%)	35	106	76	27	66 (98.5%)	6	120	81	30	0.024
**Pacemaker**	16 (18.8%)					5 (7.5%)					0.044
**Hypertension**	27 (31.8%)					17 (25.4%)					0.388

Abbreviations: n = number of patients; Min = minimum; Max = maximum; IQR = interquartile range; Perugini grade = 99mTc-DPD scintigraphy uptake based on Perugini grading system; Amyloid load = amyloid load based on the semi-quantitative scoring; IVSd = interventricular septum thickness at end-diastole; LVEF = left ventricular ejection fraction; NT-proBNP = N-terminal pro B-type natriuretic peptide; eGFR = estimated glomerular filtration rate. Troponin-T and NT-proBNP were reported in nanograms per litre (ng/L). eGFR was reported in millilitres per minute per 1.73 m² (mL/min/1.73 m²).

### Type B fibril composition with 99mTc-DPD scintigraphy uptake

Among the 67 patients with type B-fibril composition, ten (15%) had cardiac 99mTc-DPD uptake. Of these, one patient (10%) had grade 1 uptake, six patients (60%) had grade 2 uptake, and three patients (30%) had grade 3 uptake. These patients exhibited significantly greater IVSd, higher Troponin-T, and elevated NT-proBNP levels compared to type B patients without 99mTc-DPD scintigraphy uptake (*p* < 0.001 for all). Additionally, eight of the ten patients (80%) demonstrated IVSd > 12 mm. LVEF was ≥ 50% in six of the eight (75%) patients for whom this was assessable.

### Comparison between patients with type B fibrils with 99mTc-DPD scintigraphy uptake and patients with fibril Type A

As shown by Table 2, patients with type A fibrils, when compared with type B fibril patients with 99mTc-DPD uptake showed significant differences only in 99mTc-DPD uptake grade 2 (60% vs. 26%; *p* = 0.025) and grade 3 (30% vs. 71%; *p* = 0.01).”

**Table 2:** Comparison of patients with type B fibrils and positive 99mTc-DPD uptake versus type A fibrils.

Category	Fibril type B with positive DPD (*n* = 10)					Fibril type A (*n* = 85)					*p*-value
	*n* (%)	Min	Max	Median	IQR	*n* (%)	Min	Max	Median	IQR	
**Sex (male)**	8 (80.0%)					57 (67.1%)					0.405
**Age at diagnosis**	10 (100.0%)	57	77	70	14	85 (100.0%)	39	87	69	8	0.976
**Age at 99mTc-DPD**	10 (100.0%)	61	80	74.5	15	85 (100.0%)	40	87	70	8	0.416
**Perugini grade**											
Perugini grade 0	0 (0%)					0 (0.0%)					
Perugini grade 1	1 (10.0%)					3 (3.5%)					0.335
Perugini grade 2	6 (60.0%)					22 (25.9%)					0.025
Perugini grade 3	3 (30.0%)					60 (70.6%)					0.001
**Amyloid load**											
Amyloid load grade 1	3 (30.0%)					30 (35.3%)					0.739
Amyloid load grade 2	6 (60.0%)					24 (28.2%)					0.041
Amyloid load grade 3	1 (10.0%)					28 (32.9%)					0.136
Amyloid load not analysed	0 (0.0%)					3 (3.5%)					0.546
**Val30Met**	10 (100.0%)					69 (81.2%)					0.132
**IVSd**	10 (100.0%)	7	26	16	7	84 (98.8%)	7	25	16	6	0.631
IVSd > 12 mm	8 (80.0%)					73 (85.9%)					0.620
IVSd ≤ 12 mm	2 (20.0%)					11 (12.9%)					0.539
IVSd not analysed	0 (0%)					1 (1.2%)					0.73
**LVEF**											
LVEF ≥ 50%	6 (60.0%)					53 (62.4%)					0.885
LVEF 40–49%	0 (0%)					9 (10.6%)					0.279
LVEF < 40%	2 (20.0%)					9 (10.6%)					0.379
LVEF not analysed	2 (20.0%)					14 (16.5%)					0.778
**Troponin-T (ng/l)**	10 (100.0%)	9	52	26	27	80 (94.1%)	4	532	30	31	0.348
**NT-proBNP (ng/l)**	10 (100.0%)	84	7467	411	1556	84 (98.8%)	25	10,085	861.5	2581	0.286
**eGFR**	10 (100.0%)	25	94	79	25	85 (100.0%)	35	106	76	27	0.743
**Pacemaker**	1 (10.0%)					16 (18.8%)					0.491
**Hypertension**	5 (50.0%)					27 (31.8%)					0.248

Abbreviations: DPD = 99mTc-DPD scintigraphy; n = number of patients; Min = minimum; Max = maximum; IQR = interquartile range; Perugini grade = 99mTc-DPD scintigraphy uptake based on Perugini grading system; Amyloid load = amyloid load based on the semi-quantitative scoring; IVSd = interventricular septum thickness at end-diastole; LVEF = left ventricular ejection fraction; NT-proBNP = N-terminal pro B-type natriuretic peptide; eGFR = estimated glomerular filtration rate. Troponin-T and NT-proBNP were reported in nanograms per litre (ng/L). eGFR was reported in millilitres per minute per 1.73 m² (mL/min/1.73 m²).

There were no statistically significant differences in cardiac parameters, including IVSd, LVEF, Troponin-T or NT-proBNP, nor in age at diagnosis.

### Amyloid load in fat tissue biopsies

Amyloid load was analysed in 146 patients (96%). In six cases (4%), an analysable tissue biopsy could not be obtained.

Amyloid load grades in abdominal fat tissue biopsies tended to be higher in the Type B fibril group, although this did not reach statistical significance (*p* = 0.082).

Among patients with 99mTc-DPD uptake, amyloid load grade correlated with Perugini grade both within the type A fibril group (ρ = 0.391, *p* < 0.001) and across all patients with scintigraphy uptake (ρ = 0.312, *p* = 0.003). Within the type A fibril group, amyloid load grade also showed a weak but significant correlation with NT-proBNP (ρ = 0.229, *p* = 0.040). There were no statistically significant correlations between amyloid load and IVSd, Troponin-T, or NT-proBNP in any group besides the ones mentioned above.

## Discussion

The main finding in this study is the strong correlation between amyloid fibril composition in abdominal fat biopsies and clinical phenotype. This supports earlier observations^5,11^ and strengthens the hypothesis that TTR cleavage is central to the pathogenesis of ATTR cardiomyopathy^23^.

Contrary to earlier findings, a relatively large proportion (15%) of patients with type B fibrils showed pathological cardiac 99mTc-DPD uptake. These patients differed significantly from type B patients with normal cardiac 99mTc-DPD scans in parameters indicating cardiac involvement. In fact, IVSd, Troponin-T, NT-proBNP levels, and age at diagnosis in this subgroup of patients did not differ from those of type A patients. Type B ATTR fibril composition in abdominal fat tissue can thus be associated with 99mTc-DPD uptake. Amyloid fibril typing is not feasible in endomyocardial biopsies, as the material is insufficient for Westenblott analysis. Histological characterisation would require postmortem investigation. We therefore cannot fully explain these findings.

Even though the sensitivity and specificity of biopsies from subcutaneous adipose tissue in the setting of ATTR Val30Met are considered high, ^24^ it should be noted that the determination of fibril type by western blot analysis requires a substantial amount of amyloid in the tissue biopsies. Particularly in material from type A cases, the amyloid deposits are often quite limited and spotty, which can make fibril typing challenging. This limited amyloid deposition is also reflected in the semi-quantitative amyloid load grading. Sparse amyloid deposits were a more common finding in patients with type A fibrils than in those with type B fibrils. However, in patients with 99mTc-DPD uptake, we found a moderate correlation between amyloid load in fat tissue biopsies and Perugini grade, suggesting that amyloid load in adipose tissue may mirror amyloid accumulation in the heart.

A sensitive and reliable method that could be used for small amyloid deposits is warranted, and we are presently working on this problem. Theoretically, a possible explanation for the discrepancies between fibril type in adipose tissue biopsies and clinical phenotype could be an uneven distribution of fibril compositions within the adipose tissue or a distinct distribution of fibril composition between different tissues. Although there seems to be no studies in which the fibril type has been determined both in subcutaneous and heart tissue, Oshima et al. found a complete consistency in several different tissues^24^.

Moreover, the fibril type does not change over time^25^. What is still not known is whether there can be individual spots containing different fibril types in a given organ.

Furthermore, we do not really know the pathogenic relationship between ATTR Val30Met and ATTRwt amyloidosis. While the subcutaneous fat tissue deposits in the former type are usually sufficient for diagnosis and typing^26^, amyloid is often completely missing in peripheral biopsies in ATTRwt amyloidosis, despite severe cardiomyopathy. If these two diseases are different, an ATTRwt amyloidosis on top of an ATTR Val30Met type B, possibly due to susceptibility caused by seeding, would not be detected with current methods.

Finally, it is worth remembering that 99mTc-DPD uptake depends on binding to microcalcifications rather than directly to the amyloid itself^17^, whereas fibril typing reflects the biochemical composition of the TTR amyloid fibrils^7^. Despite bone scintigraphy being an excellent and widely spread diagnostic tool, we do not fully understand why only some types of amyloid cardiomyopathies demonstrate tracer uptake, whereas others do not.

Interestingly, median age at diagnosis was 55 years in the type B fibril group, suggesting that the traditional cut-off of < 50 years for early onset disease^4^ is not accurate, at least not in the Swedish population. There appears to be a significant overlap in onset ages between the two phenotypes. This finding, in combination with previous reports, ^16^ indicates that at least in some *TTR* variant populations, a negative 99mTc-DPD scintigraphy does not rule out ATTR amyloidosis, even in patients older than 50 years of age.

### Limitations

A key limitation in this study was the absence of endomyocardial biopsies, which would have allowed direct evaluation of fibril distribution in cardiac tissue. However, the material obtained from such biopsies is typically insufficient for the Western blot analysis required for fibril typing.

Another limitation of the study is that a significant number of patients (*n* = 73) were excluded due to uninterpretable or negative abdominal fat tissue biopsies, which may have affected the representativeness of the cohort. Finally, estimates of amyloid amount in the subcutaneous adipose tissue do not necessarily reflect the degree of amyloid in other organs, including the heart. Especially in patients with type A ATTR amyloidosis, the amount of amyloid deposits can be small in fat tissue despite severe cardiac involvement.

## Conclusion

In summary, the amyloid fibril type in patients with ATTRv amyloidosis correlates closely with the clinical phenotype. Our findings also suggest that 99mTc-DPD scintigraphy may better predict ATTR-CM than fibril typing alone. Moreover, in ATTR Val30Met amyloidosis, and probably in other genotypes, a normal 99mTc-DPD scan does not rule out ATTRv amyloidosis.

## Data Availability

The clinical dataset generated and analysed in this study contains personal health information and cannot be made publicly available due to legal restrictions. Data may be obtained from the corresponding author upon reasonable request.
